# Detection of bladder cancer in patients with microscopic hematuria using Oncuria-Detect: results of a prospective, multicenter international study

**DOI:** 10.1186/s12967-026-08245-4

**Published:** 2026-05-09

**Authors:** Yair Lotan, Satoshi Anai, Howard Kim, Greg Gin, Arash Akhavein, Makito Miyake, Michael Luu, Michael Ahdoot, Edward Messing, Garry Peers, Andrew Chen, Ariel Moradzadeh, Arnold I. Chin, Menghan Liu, Sunao Tanaka, Sergei Tikhonekov, Ian Pagano, Yingye Zheng, Zhen Zhang, Hideki Furuya, Charles J. Rosser

**Affiliations:** 1 Department of Urology, University of Texas Southwestern, Dallas, TX USA; 2 Seiwa Medical Center, Nara, Japan; 3 Department of Urology, Cedars-Sinai Medical Center, 110 N. George Burns Rd, Davis 2025, Los Angeles, CA 90048 USA; 4 VA Hospital Long Beach, Long Beach, CA USA; 5 Nara Medical University, Nara, Japan; 6 Samuel Oschin Comprehensive Cancer Institute, Cedars-Sinai Medical Center, Los Angeles, CA USA; 7 Department of Urology, University of Rochester Medical Center, Rochester, NY USA; 8 Aloha Urology, Honolulu, HI USA; 9 University of California Los Angeles (UCLA), Los Angeles, CA USA; 10 Fred Hutchinson Cancer Center, Seattle, WA USA; 11 Island Urology, Honolulu, HI USA; 12 Translational and Clinical Program, University of Hawaii Cancer Center, Honolulu, HI USA; 13 Cancer Prevention in Pacific Program, University of Hawaii Cancer Center, Honolulu, HI USA; 14 Department of Pathology, Johns Hopkins School of Medicine, Baltimore, MD USA; 15 Nonagen Bioscience Corp., Los Angeles, CA USA

**Keywords:** Urine, Bladder cancer, Detection, Microscopic hematuria

## Abstract

**Background:**

Microscopic hematuria occurs in up to 10% of the general population and initiates costly evaluation to ensure no bladder cancer exists. Oncuria-Detect is a 10-plex immunoassay that detects *de novo* bladder cancer by generating a protein biomarker signature from a single voided urine sample. This report details the analysis of our prospective study that compares the diagnostic performance of the multiplex Oncuria-Detect assay to that of the single-analyte (i.e., NMP22) BladderChek™ urine assay and urine cytology for identifying bladder/urothelial cancer in patients with microscopic hematuria.

**Methods:**

From September 2018 through July 2025, 9 medical facilities in the US and Japan prospectively enrolled 321 participants of whom 292 were deemed eligible. The bladder cancer diagnostic reference standard was cystoscopy with biopsy. Pre-cystoscopy, patients provided a urine sample for analysis by Oncuria-Detect and BladderChek™ (analyzed in a blinded manner) as well as urine cytology.

**Results:**

Bladder cancer was diagnosed in 22 patients (7.5%). The Oncuria-Detect assay had the following performance characteristics 82.0% sensitivity and 97.5% negative predictive value (NPV) compared to BladderChek™ (9.3% sensitivity and 95.4% NPV) and cytology (44.8% sensitivity and 97.2% NPV). Oncuria-Detect displayed favorable sensitivity for identifying early- and late-stage cancer. Oncuria-Detect had a favourable performance in detecting high-grade and MIBC (i.e., aggressive cancers); high-grade sensitivity was 93.5% (95%CI: 0.783–1.000) and MIBC sensitivity was 100.0% (95%CI: 1.000–1.000) compared to BladderChek™ high-grade sensitivity of 13.8% (95%CI: 0.000–0.370) and MIBC sensitivity was 0.0% (95%CI: 0.000–0.000) and cytology high-grade sensitivity was 60.1% (95%CI: 0.333–0.852) and MIBC sensitivity was 73.9% (95%CI: 0.000–1.000).

**Conclusions:**

In this analysis of an international prospective trial, Oncuria-Detect performed favorably in the non-invasive evaluation of bladder cancer presence in patients presenting with microscopic hematuria.

**Clinical trial number:**

Clinicaltrials.gov NCT03193541.

**Supplementary Information:**

The online version contains supplementary material available at 10.1186/s12967-026-08245-4.

## Background

Early detection is an important goal for patients at risk for bladder cancer. At presentation, approximately 75% of bladder cancer cases are non-muscle invasive bladder cancer (NMIBC), while the remaining 25% are muscle invasive bladder cancer (MIBC) or metastatic [1]. When detected early (i.e., NMIBC), the 5-year survival rate is approximately 94%, compared to at best 50% 5-year survival rate when the disease is noted to be MIBC and less than 20% 5-year survival rate when the disease is metastatic [2–4]. The most common presentation of bladder cancer is hematuria (blood in the urine) [5, 6]. Microscopic hematuria occurs in up to 10% of the general population and results in costly evaluation to ensure the microhematuria is not associated with clinically significant disease. In two large studies, 1–10% of patients with microscopic hematuria were noted to harbor bladder cancer [7–9]. Published guidelines recommend that patients with intermediate- or high-risk microscopic hematuria (Supplemental Table 1) undergo cystoscopy [10], which entails the complete visualization of the inner lining of the bladder with a small camera assessing for any tumors, a highly invasive procedure. Thus, a robust, non-invasive assay capable of further risk stratifying microscopic hematuria patients, who would truly benefit from these evaluations, is much needed.

Previously we reported the Oncuria assay [11–13], a validated non-invasive urine test based on multiplex immunoassay of 10 protein biomarkers, as an aid in the diagnosis of *de novo* bladder cancer. This assay generates a personalized bladder cancer risk profile based on a combination of the patient’s unique biomarker signature and demographic parameters. In the current prospective study, we compared Oncuria-Detect’s performance with that of BladderChek™ [14], an FDA approved immunoassay that measures bladder cancer-selective NMP22 (nuclear matrix protein 22), and urinary cytology, as aids in the diagnosis of bladder cancer in patients presenting with microscopic hematuria. Current, AUA/SUFU Guidelines on microhematuria support the role for urine markers in the evaluation of bladder cancer [15].

## Methods

### Study design

**Fig. 1:**
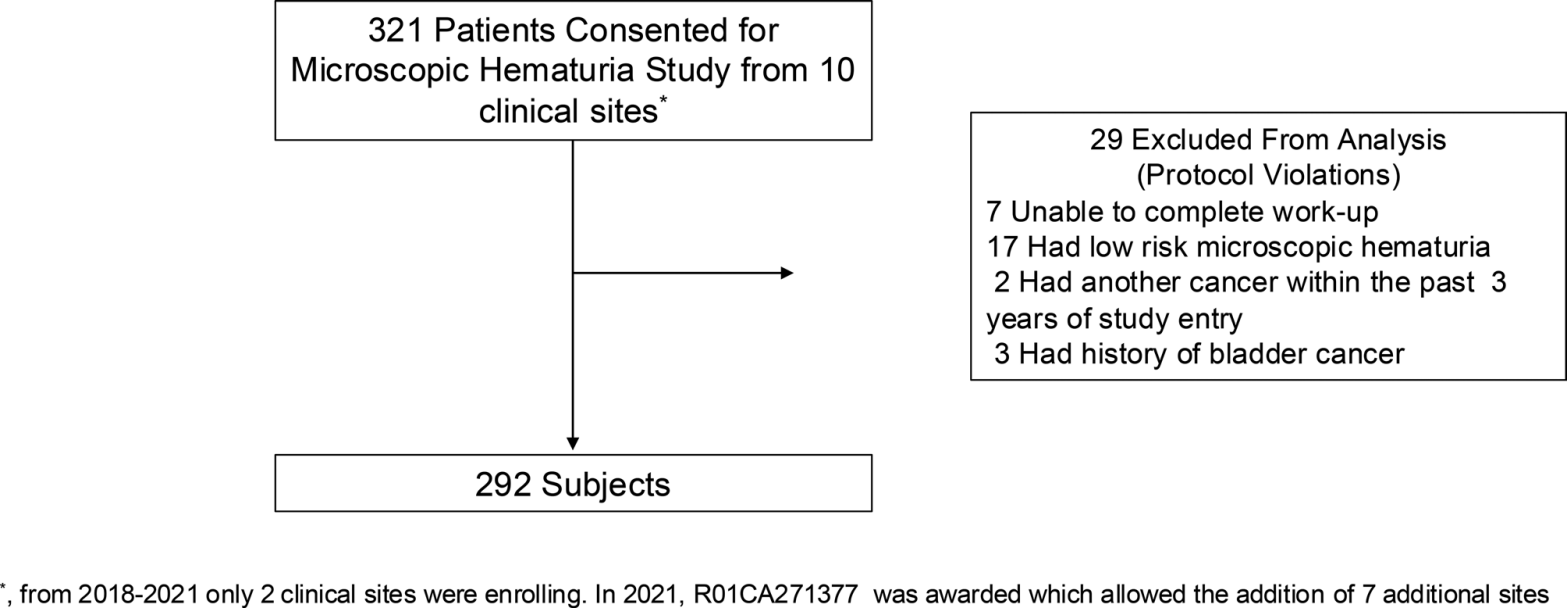
Flow diagram of study

From September 2018 through July 2025, we enrolled 321 participants of whom 292 met all inclusion/exclusion criteria and were available for analysis in this prospective longitudinal study performed at 9 clinical sites (7 in the US and 2 in Japan), including academic and private practice settings (Fig. 1 and Supplementary Table 2). All relevant institutional review boards and independent ethics committees approved the study, which was performed in accordance with the International Conference on Harmonisation Good Clinical Practice Guideline, the principles of the Declaration of Helsinki [16] and local laws and regulations. All participants provided written informed consent before participation. The trial (NCT03193541) followed the STARD 2015 and REMARK reporting guidelines [17, 18].

### Study population

The target population was adults (aged ≥ 18 years) who have documented microscopic hematuria (≥3 RBC/hpf) within 3 months of study enrollment and deemed to be intermediate or high-risk patients based on the microscopic hematuria guidelines [10] (Supplemental Table 1). We excluded participants with: (i) low-risk based on microscopic hematuria guidelines [9], (ii) gross hematuria, (iii) a history of bladder cancer; (iv) a previous cancer (excluding basal and squamous cell skin cancer and localized prostate cancer on active surveillance) within the past 3 years; (v) known active (i.e., current or ongoing) UTI, urinary retention, or stone disease (renal or bladder); (vi) renal insufficiency (creatinine ≥ 2.0 mg/dL); (vii) ureteral stents, nephrostomy tubes or bowel interposition; (viii) recent genitourinary instrumentation (within 10 days prior to signing consent); or (ix) were unable or unwilling to provide written informed consent. We did not exclude patients receiving anticoagulant and/or antiplatelet agents.

### Clinical procedures

All participants were required to provide a voided urine sample and undergo cystoscopy within 30 days after providing informed consent. All voided urine samples were collected before cystoscopy. No dietary or fluid restrictions were imposed. A portion of each urine specimen was sent to the local clinical laboratory for urinalysis. In addition, cytologic evaluation of voided urine sample or bladder barbotage and BladderChek™ testing was performed locally. Aliquots of each urine specimen allocated for Oncuria-Detect testing were briefly centrifuged to remove particulates and supernatants were frozen at −70 °C for short-term storage and shipment to the central testing facility.

Participants were considered positive for malignancy if one or more tumors or suspicious areas were observed during cystoscopy and, if removed, were defined as malignant on pathological examination. Tumors or suspicious areas seen endoscopically but not removed were considered positive for malignancy and designated as cancer stage Tx if cytology was positive/suspicious or if extensive fulguration led to complete resolution on subsequent follow-up visits. Participants were considered negative for cancer if no tumor or suspicious areas were seen endoscopically, or if tissue underwent biopsy and was defined as non-malignant on histopathological examination, or if cytology was anything other than positive/suspicious and subsequent follow-up did not note a tumor or suspicious areas. Pathological examination of biopsied tissue was performed within each institution or at a reference laboratory according to the standard practice at each facility. Staging criteria were those established by the American Joint Committee on Cancer [19].

To complete their hematuria evaluation, participants underwent radiological imaging of the genitourinary system using predominantly axial imaging (*n* = 193; CT scan = 184 and MRI = 9), ultrasound (*n* = 81), or retrograde pyelograms/intravenous pyelograms (*n* = 9). Nine participants (3%) did not undergo imaging.

### Primary and secondary outcomes

The primary outcome was the ability of the Oncuria-Detect to non-invasively detect *de novo* bladder cancer. The secondary outcome was comparing the Oncuria-Detect cancer detection rate and clinical performance with those of BladderChek™ and urinary cytology.

### Laboratory procedures

A central biorepository received all voided urine specimens for Oncuria testing. Laboratory testing was performed without knowledge of the results of cytology or clinical findings. Urine specimens were received frozen at the central testing facility and underwent a single freeze-thaw cycle before Oncuria analysis.

Oncuria®-Detect (Nonagen Bioscience Corp, Los Angeles, CA) interrogates urine samples for the presence of 10 proteins (A1AT, APOE, ANG, CA9, IL8, MMP9, MMP10, PAI1, SDC1, and VEGF) to generate a bladder cancer risk score based on the urinary protein profile. Oncuria testing was performed per manufacturer instructions.

The BladderChek Test™ (Abbott, Abbott Park, IL), an FDA-cleared point-of-care assay, was performed on fresh urine specimens at each study site following manufacturer instructions without knowledge of the results of cytology or clinical findings.

### Sample size

Assuming a cohort of 300 participants with an anticipated disease prevalence of 5%, we evaluated differences in sensitivity between Oncuria-Detect and the comparator assays, BladderChek™ and urinary cytology. Power calculations were conducted using a two-sided α of 0.05, with expected sensitivities of 80% for Oncuria-Detect and 45% for both comparator assays. Incorporating a conservative estimate of correlation between paired tests, the study was determined to provide approximately 80% power to detect the anticipated differences in sensitivity.

### Statistical analysis

The protocol enrolls consecutive participants to the extent possible, samples are collected prior to the cystoscopy, and samples are analyzed by researchers who are blinded to the clinical status of the participants, satisfying the PRoBE study design [20]. Furthermore, urologists were not privy to Oncuria-Detect nor BladderChek™ findings.

Baseline patient characteristics between cases and controls were compared using appropriate statistical tests. For continuous variables, the Wilcoxon rank-sum test was used to assess differences between groups. For categorical variables, either Fisher’s exact test or Pearson’s Chi-squared test was applied.

For Oncuria-Detect, predictions were generated for each sample using two separate models: a primary model and an aggressive model. A composite score (M~comb~) was calculated for each sample by combining the predicted probabilities from both models using a predefined algorithm [13]. Specifically, if the primary model’s predicted probability was ≤ 0.75, M~comb~ was set equal to that probability; if it exceeded 0.75, M~comb~ was set to 0.75 plus 25% of the aggressive model’s predicted probability. Classification thresholds were subsequently applied to M~comb~ to categorize samples as either positive (“Case”) or negative (“Control”). For the rule-in approach, samples with M~comb~ >0.60 were classified as positive; for the rule-out approach, a lower threshold of M~comb~ >0.12 was used.

We separately evaluated the diagnostic performance of Oncuria-Detect, BladderChek™, and cytology for the detection of urothelial cancer. Diagnostic performance was assessed for the following disease categories: urothelial cancer, NMIBC, MIBC, and low- and high-grade disease. For each test and disease category, we calculated sensitivity, specificity, positive predictive value (PPV), and negative predictive value (NPV), along with their 95% confidence intervals.

Ninety-five percent confidence intervals for sensitivity, specificity were estimated using nonparametric bootstrap resampling with 300 replicates. In each replicate, the dataset was resampled with replacement, and the relevant statistics were recalculated. The 2.5th and 97.5th percentiles of the bootstrap distribution were used to define the confidence intervals.

All statistical analyses were performed using R statistical software (version 4.5.1; R Foundation for Statistical Computing, Vienna, Austria) [21]. All tests were two-sided, and a *p*-value < 0.05 was considered statistically significant.

## Results

Over a seven-year period, which included the COVID pandemic, we recruited 321 participants of whom 292 were eligible for analysis. Demographic and baseline characteristics of the participants along with risk factors of bladder cancer are summarized in Table 1. Among the 292 participants who had hematuria evaluation, 22 (7.5%) had bladder cancer. No patient was found to have upper tract urothelial carcinoma. The median age of the participants with carcinoma was 69 years (interquartile range, 65–78 years), having a higher percentage of men vs. women (82% vs. 18%), a higher percentage self-identifying as Asian vs. Caucasian (59% vs. 41%), a higher percentage reported former/current tobacco use vs. never tobacco use (72.7% vs. 47.6%), and a higher percentage of AUA microhematuria high risk vs. intermediate risk (91% vs. 9%). Compared to participants negative for carcinoma, those with bladder cancer were predominantly male (*p* = 0.014) and had a history of tobacco use (*p* = 0.009).

**Table 1:** Study participants characteristics

Characteristic	Overall *N* = 292^*1*^	Non-bladder cancer *N* = 270^*1*^	Bladder cancer *N* = 22^*1*^	**p-value**^*2*^
**Age**	68 (58, 75)	67 (58, 75)	69 (65, 78)	0.077
**Hispanic**				0.7
No	262 (90%)	240 (89%)	22 (100%)	
Refused	3 (1.0%)	3 (1.1%)	0 (0%)	
Unknown	6 (2.0%)	6 (2.2%)	0 (0%)	
Yes	21 (7.2%)	21 (7.7%)	0 (0%)	
**Race**				0.10
White	138 (47%)	129 (48%)	9 (41%)	
Black	33 (11%)	33 (12%)	0 (0%)	
Asian	102 (35%)	89 (33%)	13 (59%)	
Hispanic	0 (0%)	0 (0%)	0 (0%)	
Pacific Islander	3 (1%)	3 (1%)	0 (0%)	
Other	16 (6%)	16 (6%)	0 (0%)	
**Sex**				0.014
Male	166 (57%)	148 (55%)	18 (82%)	
Female	126 (43%)	122 (45%)	4 (18%)	
**Tobacco Ever**				0.009
Never smoker	145 (49.7%)	140 (51.9%)	5 (22.7%)	
Former smoker	103 (35.3%)	89 (33.0%)	14 (63.6%)	
Current smoker	36 (12.3%)	34 (12.6%)	2 (9.1%)	
Unknown	8 (2.7%)	7 (2.6%)	1 (4.5%)	
Pack Years	19 (10, 30)	19 (10, 30)	20 (5, 36)	0.8
Unknown	151	146	5	
**AUA Microhematuria Risk Classification**				0.045
High risk	212 (73%)	192 (71%)	20 (91%)	
Intermediate risk	80 (27%)	68 (29%)	2 (9%)	

^*1*^ Median (Q1, Q3); *n* (%)

^*2*^ Wilcoxon rank sum test; Fisher’s exact test; Pearson’s Chi-squared test

All tumors seen during cystoscopy were excised and assessed for tumor grade and stage. Among the 22 patients with urothelial carcinoma, 18 (82%) were NMIBC (stages Ta, Tis, or T1), and 4 (18%) were MIBC (≥T2). No participants had detectable metastases or involvement of regional lymph nodes. Of these 22 tumor specimens, 7 (32%) were low-grade and 15 (68%) were high-grade.

BladderChek™ test results were available for 292 participants, while urine cytology results were available for 285 participants (7 or 2.4% missing) in which 8 specimens were reported as inadequate due to insufficient cellularity. Cystoscopic findings were indeterminate in 4 participants of whom 3 had these indeterminant lesions biopsied and were noted to be benign. Both cytology and BladderChek™ were negative in these 4 participants. Overall, cystoscopy alone failed to detect 1 of the 22 confirmed malignancies (positive cytology led to this participant to be biopsied).

Table 2 illustrates the performance of Oncuria-Detect, BladderChek™ and cytology in this cohort. Oncuria-Detect had a sensitivity of 82.0% (95%CI: 0.651–0.955) at a specificity of 37.8% (95%CI: 0.315–0.435), and an NPV of 97.5% (95%CI: 0.952–0.994), and PPV of 6.5% (95%CI: 0.051–0.077). BladderChek™ had a sensitivity of 9.3% (95%CI: 0.000–0.250) at a specificity of 99.6% (95%CI: 0.989–1.000), and an NPV of 95.4% (95%CI: 0.950–0.962), and PPV of 61.3% (95%CI: 0.000–1.000), whereas cytology was noted to have a sensitivity of 44.8% (95%CI: 0.227–0.634) at a specificity of 99.3% (95%CI: 0.982–1.000), and an NPV of 97.2% (95%CI: 0.961–0.981), and PPV of 77.8% (95%CI: 0.508–1.000).

**Table 2:** Performance of Oncuria-Detect, BladderChek™ and cytology for urothelial cancer detection

	TP	FN	FP	TN	Total	Sensitivity	Specificity	PPV	NPV
**Oncuria-Detect**									
Urothelial cancer	18	4	167	103	292	0.820 (0.651, 0.955)	0.378 (0.315, 0.435)	0.065 (0.051, 0.077)	0.975 (0.952, 0.994)
High-grade	14	1	171	106	292	0.935 (0.783, 1.000)	0.379 (0.321, 0.434)	0.074 (0.061, 0.083)	0.991 (0.971, 1.000)
Low-grade	4	3	181	104	292	0.575 (0.133, 1.000)	0.362 (0.302, 0.413)	0.045 (0.011, 0.075)	0.942 (0.881, 1.000)
Stage T2, T3	4	0	181	107	292	1.000 (1.000, 1.000)	0.368 (0.311, 0.421)	0.077 (0.071, 0.083)	1.000 (1.000, 1.000)
Stage Ta, Tis, T1	14	4	171	103	292	0.779 (0.554, 0.944)	0.372 (0.309, 0.425)	0.061 (0.043, 0.075)	0.970 (0.935, 0.992)
**BladderChek™**									
Urothelial cancer	2	20	1	269	292	0.093 (0.000, 0.250)	0.996 (0.989, 1.000)	0.613 (0.000, 1.000)	0.954 (0.950, 0.962)
High-grade	2	13	1	276	292	0.138 (0.000, 0.370)	0.996 (0.989, 1.000)	0.668 (0.000, 1.000)	0.956 (0.950, 0.968)
Low-grade	0	7	3	282	292	0.000 (0.000, 0.000)	0.989 (0.976, 1.000)	0.000 (0.000, 0.000)	0.949 (0.949, 0.950)
Stage T2, T3	0	4	3	285	292	0.000 (0.000, 0.000)	0.989 (0.976, 1.000)	0.000 (0.000, 0.000)	0.949 (0.949, 0.950)
Stage Ta, Tis, T1	2	16	1	273	292	0.112 (0.000, 0.294)	0.996 (0.989, 1.000)	0.641 (0.000, 1.000)	0.955 (0.950, 0.964)
**Cytology**									
Urothelial cancer	10	12	2	268	292	0.448 (0.227, 0.634)	0.993 (0.982, 1.000)	0.778 (0.508, 1.000)	0.972 (0.961, 0.981)
High-grade	9	6	3	274	292	0.601 (0.333, 0.852)	0.990 (0.978, 1.000)	0.765 (0.535, 1.000)	0.979 (0.966, 0.992)
Low-grade	1	6	11	274	292	0.129 (0.000, 0.423)	0.962 (0.938, 0.983)	0.138 (0.000, 0.417)	0.955 (0.947, 0.969)
Stage T2, T3	3	1	9	279	292	0.739 (0.000, 1.000)	0.969 (0.951, 0.990)	0.546 (0.000, 0.827)	0.986 (0.949, 1.000)
Stage Ta, Tis, T1	7	11	5	269	292	0.382 (0.133, 0.586)	0.982 (0.967, 0.996)	0.539 (0.253, 0.830)	0.968 (0.956, 0.978)

Notably, Oncuria-Detect had a favourable sensitivity in detecting low-grade and high-grade tumors as well as NMIBC and MIBC. Low-grade sensitivity was 57.5% (95%CI: 0.133–1.000), high-grade sensitivity was 93.5% (95%CI: 0.783–1.000), while NMIBC sensitivity was 77.9% (95%CI: 0.554–0.944) and MIBC sensitivity was 100.0% (95%CI: 1.000–1.000). BladderChek™ demonstrated low-grade sensitivity of 0.0% (95%CI: 0.000–0.000), high-grade sensitivity of 13.8% (95%CI: 0.000–0.370), while NMIBC sensitivity was 11.2% (95%CI: 0.000–0.294) and MIBC sensitivity was 0.0% (95%CI: 0.000–0.000). Cytology was noted to have low-grade sensitivity of 12.9% (95%CI: 0.000–0.423), high-grade sensitivity was 60.1% (95%CI: 0.333–0.852), while NMIBC sensitivity was 38.2% (95%CI: 0.133–0.586) and MIBC sensitivity was 73.9% (95%CI: 0.000–1.000) (Table 2).

## Discussion

The non-invasive Oncuria-Detect multiplex bladder cancer assay displayed favorable overall sensitivity (82.0%) for identifying neoplastic disease in patients with microscopic hematuria. Oncuria-Detect maintained its favorable sensitivity in identifying low-grade and early stage cancers, when treatment has the highest likelihood of success [2–4], as well as favorable sensitivity for capturing more advanced disease. The specificity of Oncuria-Detect in patients with microscopic hematuria (37.8%) was lower than the specificity of BladderChek™ (99.65) and cytology (99.3%). The specificity of each of the three comparator tests was not impacted by coexisting inflammation, urolithiasis, or urinary tract infection (*data not shown*).

An earlier Oncuria-Detect validation study demonstrated 93% sensitivity and 99% negative predictive value (NPV) for identifying bladder cancer in 362 patients with hematuria (gross or microscopic) [12]. The current study’s high NPV with Oncuria-Detect (97.5%) was similar to that with both BladderChek™ (95.4%) and cytology (97.2%). The high NPV values are largely driven by the low prevalence of bladder cancer in the cohort. Given the low sensitivities of BladderChek™ and cytology, reliance on NPV alone in this setting would be misleading, as both tests fail to identify a substantial proportion of true positives. Because only 1–10% of patients with microscopic hematuria have bladder cancer [7–9], ancillary diagnostic approaches with a demonstrably high NPV and high sensitivity may confidently rule out disease presence and thereby reduce the need for performing costly and invasive follow-on cystoscopies [10].

In a similar cohort comprised of only patients with microscopic hematuria, 22 tumors were found in 270 eligible patients evaluated with CxBladder-Triage [22]. Both CxBladder-Triage and Oncuria-Detect demonstrated comparable sensitivities for detecting urothelial carcinoma, particularly for high-grade disease, although Oncuria-Detect exhibited slightly lower overall sensitivity (82.0% vs. 90% for all urothelial carcinoma). Specificity was generally higher for CxBladder-Triage 56% overall compared with Oncuria-Detect 37.8%; while both assays maintained high negative predictive values (NPV > 97%), reflecting a strong potential for ruling out disease in low-prevalence populations. Positive predictive values (PPV) were low for both tests, consistent with the low prevalence of urothelial carcinoma in the study populations, highlighting that a positive result should be interpreted in the context of clinical risk factors. Overall, these findings suggest that while both assays are effective at identifying patients with disease, differences in specificity and PPV may influence clinical decision-making depending on the intended use setting.

Cystoscopy is an excellent tool for directly inspecting the bladder lining. However, procedural accuracy can be compromised by poor visualization due to inflammation or bleeding, and flat lesions such as carcinoma in situ (CIS or TIS) may be difficult to discriminate from normal bladder tissue using standard white-light cystoscopy [23]. In fact, cystoscopy alone failed to detect 1 of the 22 (4.5%) confirmed malignancies, which had a positive cytology and a positive Oncuria test. These limitations of cystoscopy make non-invasive urine-based assays that can quickly and accurately identify and characterize bladder cancer status especially advantageous. Growing evidence suggests that cystoscopy may be overemployed, increasing both treatment costs and risks [24]. In a 2025 report that evaluated Oncuria-Detect in an international real-world population with hematuria (microscopic and gross), the authors concluded that Oncuria-Detect’s high NPV may effectively preclude a significant number of patients from requiring subsequent cystoscopy [13]. In the current study, 35% of the participants could be reliably ruled out of harboring cancer. In the US alone, this would save hundreds of millions of healthcare dollars annually while avoiding potential harms associated with performing unnecessary procedures [25].

Multiplex biomarker signatures (nucleic acid or protein) have emerged as powerful oncology diagnostic resources for evaluating cancers of diverse anatomical origin, e.g., the breast [26], prostate [27], and colon [28]. In recent years, multiplex urine-based tests have been launched into the clinic as laboratory-developed tests (LDTs). These include DNA-based tests (e.g., AssuredMDx, UroAmplitude, Uromonitor-V2) and RNA-based tests (e.g., CxBladder, Xpert Bladder Cancer Detection, BladderCARE) [29]. Less attention has been given to protein-based assays [30]. Advantages of multiplex immunoassays over evaluating multiple analytes individually include increased efficiency at reduced cost, the potential to map disease-associated proteomic networks, and compatibility with modern high-throughput automated instrumentation platforms [31].

Oncuria-Detect calculates a bladder cancer risk score by evaluating a 10-protein biomarker profile in voided urine specimens, which allows reliable discrimination between patients with neoplastic disease and unaffected individuals [11–13]. Variations of the Oncuria algorithm have also been engineered and patient-personalized to predict bladder cancer responsiveness to treatment, track progress, and monitor for disease recurrence.

A primary limitation of this study is the modest sample size, including only 22 bladder cancer events, which limits statistical power, particularly for subgroup analyses by stage and grade. Consequently, certain estimates—such as the observed 100% sensitivity for muscle-invasive disease—should be interpreted with caution given the small number of events. The low event rate also affects the precision of positive predictive value estimates. In addition, cytology and pathology were assessed locally, introducing potential inter-center variability. The study was initiated during the COVID-19 pandemic, which delayed site activation and enrollment, and as an NIH-funded study without industry support, accrual has been slower than in comparable industry-sponsored trials. Enrollment is ongoing and will continue until 900 participants or 100 bladder cancer events are reached.

Despite these limitations, the cohort reflects a clinically relevant population undergoing evaluation for microscopic hematuria, with a bladder cancer prevalence of 7.5%, consistent with prior reports (1–10%) [7–9]. The findings are therefore generalizable to similar populations, including older adults with a substantial proportion of current or former tobacco users.

## Conclusions

The multiplex Oncuria-Detect assay displayed favorable performance for identifying bladder cancer in patients with microscopic hematuria. Oncuria-Detect’s high sensitivity and NPV supports its use as a rapid, non-invasive, adjunctive approach to supplement gold-standard diagnostic approaches such as cystoscopy in diagnosing bladder cancer. Additional studies are underway to confirm these results.

## Electronic supplementary material

Below is the link to the electronic supplementary material.

Supplementary Material 1

Supplementary Material 2

## Data Availability

De-identified original datasets are available from the Corresponding Author for *bona fide* researchers, upon reasonable request at the conclusion of the study.

## Abbreviations

NPV: Negative predictive value
PPV: Positive predictive value
NMIBC: Non-muscle invasive bladder cancer
MIBC: Muscle invasive bladder cancer
STARD: Standards for Reporting of Diagnostic Accuracy Studies)
UTI: Urinary tract infection
A1AT: SerpinA1
ANG: Angiogenin
APOE: Apolipoprotein
CA9: Carbonic Anhydrase 9
IL8: Interleukin 8
MMP9: Matrix Metalloproteinase 9
MMP10: Matrix Metalloproteinase 10
PAI1: Plasminogen Activator Inhibitor 1/SerpinE1
SDC1: Syndecan 1
VEGF: Vascular Endothelial Growth Factor
PRoBE: Prospective-specimen-collection, retrospective-blinded-evaluation
