# Supplementary material for: Detection of bladder cancer in patients with microscopic hematuria using Oncuria-Detect: results of a prospective, multicenter international study
Source: J Transl Med. 2026 May 9;24:791. doi: 10.1186/s12967-026-08245-4 (PMC13281342; doi:10.1186/s12967-026-08245-4)
Supplement: Supplementary file 1 — Supplementary Material 1 [file 12967_2026_8245_MOESM1_ESM.docx]

**Supplemental Table 1 AUA/SUFU Microscopic hematuria risk stratification system 2025**

| **Risk of cancer** | **Low/negligible (0%-0.4%)** | **Intermediate**  **(0.2%-3.1%)** | **High (1.3%-6.3%)** |
| --- | --- | --- | --- |
| Number of criteria to meet | All | One or more | One or more |
| Degree of hematuria on a single urinalysis | 3-10 RBC | 11-25 RBC/hpf | >25 RBC/hpf |
| Alternative criteria for degree of hematuria |  | Previously low/negligible-risk patient with no prior evaluation and 3 to 25 RBC/hpf on repeat urinalysis | History of gross hematuria |
| Age for women | <60 years | >60 years | Women should not be categorized based on age |
| Age for men | <40 years | - 1. years | > 60 years |
| Smoking history | Never smoker or < 10 pack years | - 1. pack years | > 30 pack years |
| Presence of additional risk cancer risk factors | None | Any | One or more plus any high-risk features |

Adapted from ref 10

RBC = red blood cells, hpf = high power field
