# Supplementary material for: Detection of bladder cancer in patients with microscopic hematuria using Oncuria-Detect: results of a prospective, multicenter international study
Source: J Transl Med. 2026 May 9;24:791. doi: 10.1186/s12967-026-08245-4 (PMC13281342; doi:10.1186/s12967-026-08245-4)
Supplement: Supplementary file 2 — Supplementary Material 2 [file 12967_2026_8245_MOESM2_ESM.docx]

**Supplemental Table 2 Participating sites and their activity**

1. Aloha Urology, Honolulu, HI, USA – 16 participants
2. Department of Urology, Cedars-Sinai Medical Center, Los Angeles, CA, USA – 78 participants
3. Island Urology, Honolulu, HI, USA – 31 participants
4. Nara Medical University, Nara, Japan – 22 participants
5. Seiwa Medical Center, Nara, Japan – 31 participants
6. University of California Los Angeles (UCLA), Los Angeles, CA – 07 participants
7. Department of Urology, University of Rochester, Rochester, NY – 34 participants
8. Department of Urology, University of Texas Southwestern, Dallas, TX, USA – 50 participants
9. VA Hospital Long Beach, Long Beach, CA, USA – 23 participants

**TOTAL 292**
